# Quartz Crystal Microbalance-Based Aptasensors for Medical Diagnosis

**DOI:** 10.3390/mi13091441

**Published:** 2022-09-01

**Authors:** Semra Akgönüllü, Erdoğan Özgür, Adil Denizli

**Affiliations:** Department of Chemistry, Division of Biochemistry, Hacettepe University, Ankara 06800, Turkey

**Keywords:** aptamer, aptasensor, biosensor, label-free detection, diagnosis, medical applications, quartz crystal microbalance

## Abstract

Aptamers are important materials for the specific determination of different disease-related biomarkers. Several methods have been enhanced to transform selected target molecule-specific aptamer bindings into measurable signals. A number of specific aptamer-based biosensors have been designed for potential applications in clinical diagnostics. Various methods in combination with a wide variety of nano-scale materials have been employed to develop aptamer-based biosensors to further increase sensitivity and detection limit for related target molecules. In this critical review, we highlight the advantages of aptamers as biorecognition elements in biosensors for target biomolecules. In recent years, it has been demonstrated that electrode material plays an important role in obtaining quick, label-free, simple, stable, and sensitive detection in biological analysis using piezoelectric devices. For this reason, we review the recent progress in growth of aptamer-based QCM biosensors for medical diagnoses, including virus, bacteria, cell, protein, and disease biomarker detection.

## 1. Introduction

Aptamers have attained popularity as a promising molecular recognition element bearing values of the dissociation constant (K_d_) from picomolar to nanomolar ranges in sensing applications such as environmental safety, food safety, and healthcare [1,2,3,4,5]. Aptamers were first reported in 1990 [6]. They are short-chain oligonucleotides of RNA or single-stranded DNA commonly chosen via in vitro operation, namely, systematic evolution of ligands by exponential enrichment (SELEX) [7]. As such, they are described as artificial antibodies due to their high affinity and high-resolution molecular discrimination to their target analytes [8]. Unlike antibodies, aptamers are formed through chemical synthesis, and compared with natural antibodies their high surface density leads to less steric hindrance, enhancing their efficiency in recognizing targets [9]. In addition, aptamers maintain their properties during storage at room temperature and in different reactionary environments, are readily labeled with various reporters [3,10], and costs for their synthesis are relatively low, which is an important feature in diagnostic analysis [11]. A diverse type of aptamer has been reported to recognize targets such as metal ions [12], small organic compounds [13], toxins [14], nucleotides [15], peptides [16], amino acids [17], enzymes [18], proteins [19], hormones [20], bacteria [21], and whole cells [22] with high sensitivity and affinity.

**Figure 1:**
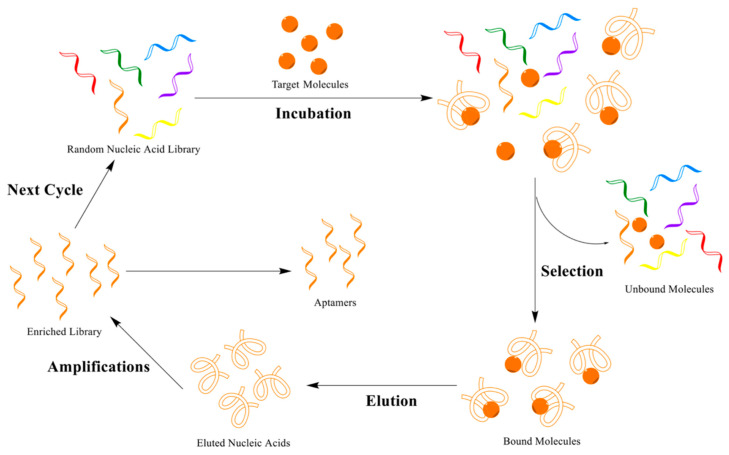
Process of SELEX technology. Reprinted with permission from Ref. [31] 2021, Liu.

Aptamer biomolecules consist of short single-stranded DNA/RNA nucleic acid/ oligonucleotide sequences chosen from random nucleic acid libraries by a SELEX process [23,24,25,26,27]. Nucleic acids are more resistant to changes in physical conditions, including pH differences and high temperature, than proteins, and they have smaller sizes than antibodies. In addition, chemical modifications can be made easily. Finally, when considering obtaining antibodies, the preparation of aptamers has several advantages including quicker synthesis [28,29]. Aptamers are one of the most employed biorecognition materials for proteomics, medical diagnostics, and applications. They are of great interest as an alternative biomolecular recognition element to create an ideal detection system via rapid and robust response, high specificity, sensitivity and biostability, and cost-effectiveness. Aptamers selected and designed with SELEX technology are short single nucleic acid sequences with fascinating features involving recognition of their target analytes. In general, the process of the SELEX technique contains four steps (Figure 1): (i) incubation with target; (ii) selection; (iii) elution of aptamers; and (iv) amplification of eluted aptamer. First, aptamers are chosen from a large range of random nucleic acid libraries. After a few selection steps, aptamers become enriched for binding to their related molecule with high specificity and affinity [30].

Herein, unique features such as higher chemical stability, easy low-cost mass manufacturability, and longer self-time compared to natural biorecognition elements, namely, antibodies, enzymes, and proteins, enable the use of aptamers in biosensors as excellent biorecognition elements in sensing applications [7]. Biosensors are analytical tools employed to detect target analytes in biological or chemical reactions via measurable signals proportional to the analyte level in an interested medium in a wide range of concentrations at the ng/mL or even fg/mL level. They combine a biorecognition material with a chemical, physical, or physicochemical transducer [32,33,34,35,36]. The target analytes may be of various kinds, including ions [37], gases [38], drugs [39], oligonucleotides [40], or proteins [41], and there may be different kinds of transducer as well, such as plasmonic-optical [42], mass-sensitive piezoelectric [43], thermal calorimetric thermometric [44], and chemical–electrochemical–electrical [45]. Biosensors offer comparable sensitivity and selectivity while enabling online monitoring and real-time detection [46,47]. In recent years, use of such sensors has played a noteworthy role in critical research topics [48]. Different types of aptamer-based biosensors, namely, aptasensors, are classified differently according to their transduction mechanisms, including electrochemical, optical, field effect transistor, calorimetric, and piezoelectric [5,49,50,51]. A quartz crystal microbalance as a mass-sensitive based sensor (QCM) establishes the mass per unit area by measuring the change in frequency (Δƒ), which is related to the mass accumulated on the quartz crystal resonator electrode [52]. The rapid, accurate, real-time, label-free, and even on-site detection capability of the QCM technology, and especially its high sensitivity, have gained attention in the design of novel diagnostic tools integrating the significant advantages of aptamers [53]. In this review, we briefly point out several typical aptamer biosensors used for medical diagnostic analysis. We focus on introducing novel methods for improving the potential of current analytical sensing approaches in terms of sensitivity and specificity.

## 2. QCM Biosensor

**Figure 2:**
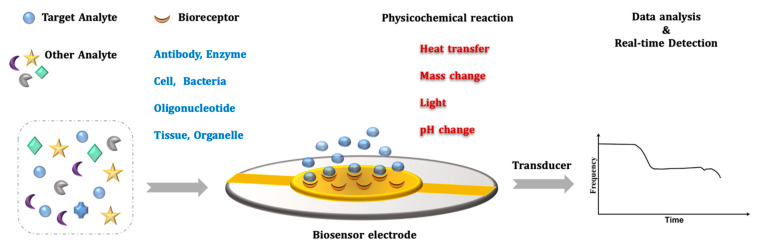
Working principle of a biosensor.

Biosensors are tools that detect and determine specific target molecules and convert recognition of target molecules into the measurable signals [42,54,55]. This detectable signal is easily induced by specific molecular interactions between target molecules and recognition materials (i.e., receptors) [56]. Typically, a biosensor contains two major units, a receptor and a transducer, as shown in Figure 2. The optimal recognition material must be highly sensitive and specific for the target analyte of interest. It should adequately recognize and specifically capture the target analyte, resulting in a quick reply and strong performance. Recognition elements, including nucleic acids, antibodies, cells, and enzymes, can now be easily produced in an experimental laboratory. A transducer converts the biomolecular binding events into measurable signals, including optical and electrochemical signals as well as mass changes [57,58]. The label-free surface-sensitive technique is perfect for monitoring interaction processes in liquid samples without the need to label related molecules, as they only generate signals by their physical existence on the recognition surface [59]. In addition to the low cost of the label-free technique, it offers the capability of detecting the kinetic behavior of biological interactions even at the submolecular level, providing real-time monitoring [60]. Several label-free transduction-based studies have been reported and have proven suitable in medical areas such as the pharmaceutical industry or healthcare for point-of-care testing and applications in basic research [61].

The piezoelectric crystal materials of an electromechanical transducer are fitted for employment as biosensors and actuators in devices and structures [62,63,64]. These piezoelectric devices provide quick and label-free detection of the target molecules, resulting in specific and/or non-specific binding on the resonator surface. QCM biosensors are an effective analytical platform, and have been broadly applied to monitor interactions between biomolecules [65,66,67,68]. Compared to traditional methods, they provide label-free and real-time detection, easy use with modern technologies, portable size, high sensitivity, low cost, and basic data analysis [69,70,71,72]. QCM is nanogram-sensitive, and physical technologies can determine changes in resonance frequency (Δ*f*) of the electrically driven quartz crystal by changes in thickness or mass per unit field (Δ*m*). A relationship between the mass loaded on the quartz crystal surface and the resonant frequency is derived by the Sauerbrey Equation (1):

According to Sauerbrey effect [73,74],

(1) $$\Delta f=-\frac{2{f_{0}}^{2}}{A\sqrt{\rho_{q}\mu_{q}}}\Delta m$$

where *f*_0_ is the resonant frequency, Δ*f* is the change in resonant frequency (Hz) due to mass loaded per unit area (Δ*m*) on the surface, ρ is the quartz density (2.648 g/cm^3^), and μ is the shear modulus of quartz (2.947 × 10^11^ g·cm^−1^·s^2^).

**Figure 3:**
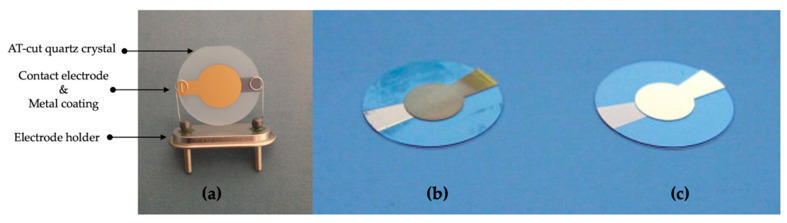
A quartz crystal with gold electrodes: (**a**) typical AT-cut piezoelectric crystal coated with two gold electrodes, one on each side; reprinted with permission from Ref. [75] 2015, Bragazzi. Modified gold electrode (**b**) and bare gold surface (**c**) of AT-cut quartz with keyhole electrode. Reprinted with permission from Ref. [105] 2010, Biemmi.

Quartz is the acoustic resonator and experiences the piezoelectric effect that induces acoustic waves by applying an alternating current to the quartz crystal. The QCM is known as a thickness shear mode resonator or bulk acoustic wave transducer [75]. A typical AT-cut quartz crystal with gold electrode photographs is shown in Figure 3. The sole design criterion of thickness–shear mode resonators for frequency control is frequency stability. The AT-cut is most suitable [76,77]. AT-cut quartz crystals are typically employed as sensor components, although the needs of sensor applications are more complicated [78,79]. The full physical definition of a viscoelastic charge in contact with a quartz crystal resonator has allowed the study of the mechanical characteristics of different materials coated on the sensor surface, such as the viscoelastic characteristics of polymers. The “acoustically thin” or “acoustically thick” coatings are of main significance [76,78]. AT-QCM sensors are becoming a good alternative analytical technique in numerous applications. They are widely used as QCM sensors in gaseous mediums [80,81]. Following the first studies showing that QCM can be used for the liquid phase, the use of crystal resonators has been reported for a great number of applications in various areas [82]. Biorecognition materials undergo important changes in physical or chemical features in response to surrounding stimuli, including solvents, temperature, pH, magnetic fields, chemical agents, and electrical fields. Recently, pH-responsive materials have increasingly been used in different fields [83,84,85].

On the other hand, QCM technology provides use to the physical parameters of the sample by measuring the dissipation factor or another equivalent electrical parameter [86]. Quartz crystals have been broadly employed to analyze mass, molecular interaction, membrane structure, and viscoelasticity changes on the surface of the electrodes [87]. Viscoelastic and conformational properties of the sample are monitored depending on the dispersion parameter [86]. QCM with dissipation monitoring (QCM-D) is a powerful device employed to sensitively analyze the real-time and label-free responses of polymer films to external responses. The QCM-D technique is widely utilized to monitor film growth, material adsorption, thin film swelling, and ion exchange. QCM-D, similar to a QCM tool, utilizes the inverse piezoelectric effect, which results in vibrational oscillations of a quartz crystal when an alternating potential is applied. The difference between these two methods is that QCM-D measures the change in both the resonant frequency (Δ*f*) and the dissipation of oscillations (Δ*D*), while QCM only measures the change in the resonant frequency [84,88,89]. QCM-D has the ability to sensitively monitor mass changes on small time scales [90]. Recently, there have been many reports of the QCM-D technique being employed as a powerful device to understand a variety of phenomena such as fouling [91], swelling [92], adsorption [93], and ion exchange [94].

QCM is a highly nanogram-sensitive technology of mass variations on the electrode surface. In this technique, a specific bioreceptor (for example, an antibody or aptamer) for the target biomolecule can be attached to the electrode surface. Thus, bioreceptors on the QCM electrode surface can interact with target molecules, which can be detected as a result of the detectable frequency changes [28]. Several methods can be applied to the design of QCM-based biosensor surfaces for various application areas. Many uses of QCM biosensors have been published for detection of various molecules, such as amino acids [95], proteins [96], enzymes [97], drugs [98], vitamins [99], metals [100], pesticides [101], biomarkers [102], antibiotics [103], bacteria [104], etc. Mass-sensitive QCM biosensors are commonly utilized for the detection of biomarkers.

It is very important to utilize accurate and timely diagnostic methods to prevent the progress of a disease and stop the chain of transmission through early detection. Traditional devices are often time-consuming and costly. It is necessary to develop clinically sensitive, quick, and cost-effective clinical diagnostic methods. QCM biosensors are one of these technologies. QCM technologies are used as strong sensing devices because of their label-free properties, which provide the detection and determination of a large variety of biomolecules [53]. It is quite useful to combine aptamers with QCM as a transducer. The relatively small size of aptamers is a positive advantage for mass-based sensing devices and other transducer applications [106]. QCM devices have been broadly employed in several fields [107], such as analytical chemistry [108], immunology [109], and drug development [110], because of their high-quality features and high sensitivity [111]. The following critical review of recent progress on QCM aptasensors for the medical diagnosis is intended to present researchers with a detailed understanding of their development and design while providing useful foundations for further practical biomedical applications.

## 3. Application of QCM Aptasensors for Medical Diagnostics

This section reviews QCM aptasensors for the selective recognition and detection of various biological molecules such as viruses, bacteria, proteins, and cells. In addition, immobilization techniques of aptamers, preparation of QCM detection electrodes/chips, and performance in terms of detection limit, selectivity, and sensitivity are examined.

### 3.1. Viruses

Viruses are pathogenic microorganisms that are the reason for many infectious diseases. Viruses can live and multiply in the human body and spread easily and rapidly from infected people to healthy people. Therefore, timely detection of disease-causing viruses is the most important way to prevent the unwanted spread of infectious diseases and ensure timely medical treatment [107]. Aptamers are produced for a broad variety of viruses such as EBOV, HIV, HBV, severe acute respiratory syndrome (SARS), influenza viruses. dengue virus, rabies virus, norovirus, and vaccinia virus.

**Figure 4:**
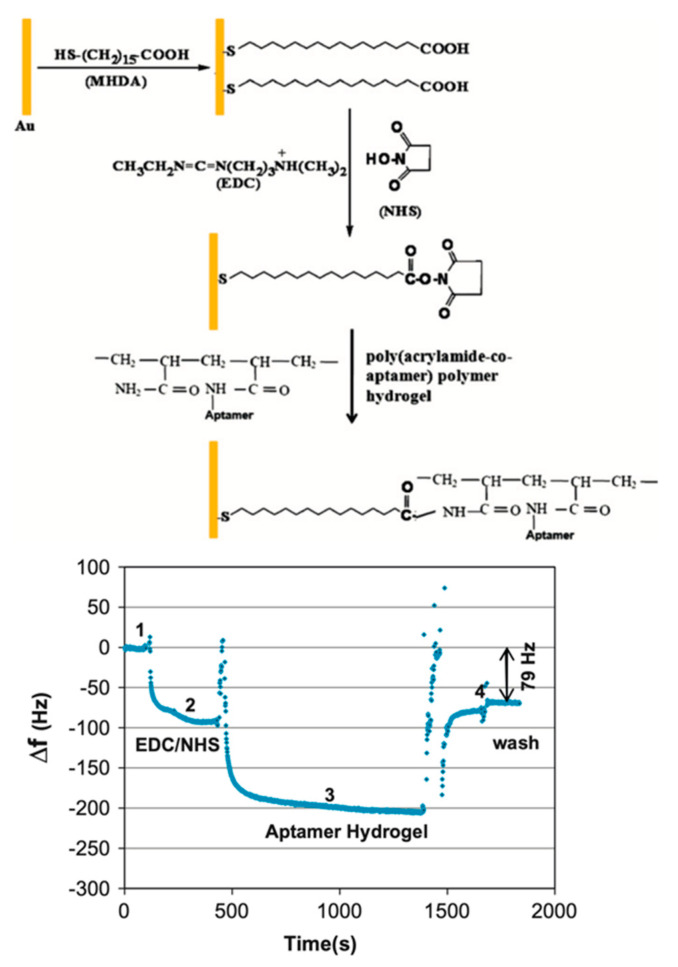
Preparation of aptamer hydrogel-coated QCM aptasensor and QCM sensorgram: (1) SAM created on the sensor in equilibrium with distilled water; (2) NHS/EDC activation; (3) poly(acrylamide) co-aptamer polymer hydrogel immobilization; (4) washing to obtain the baseline. Reprinted with permission from Ref. [114] 2013, Wang.

Avian influenza viruses (AIV) have created worldwide concern because of their potential pandemic threat to public health and major economic losses [112,113]. Wang et al. developed an ssDNA crosslinked polymeric hydrogel-coated QCM aptasensor for quick and selective detection of AIV H5N1. The chosen specific aptamer and single-stranded DNA (ssDNA) were used to form the crosslinker in a polymeric hydrogel. The aptamer-attached polymeric hydrogel was coated homogeneously onto a QCM electrode’s gold surface using a self-assembled monolayer (SAM) technique. The different molar ratios of three polymeric hydrogels were synthesized with acrylamide and aptamer. The hydrogel swelling was monitored with a QCM device of decreasing frequency. The authors reported that the 1:1 hydrogel-aptamer coated QCM electrode provided the best sensitivity. The limit of detection (LOD) was found to be 0.0128 HAU. The detection time for detection of AIV H5N1 was only 30 min with the designed aptamer-attached hydrogel-coated QCM aptasensor [114]. The preparation of this QCM sensor design and sensorgram is shown in Figure 4.

**Figure 5:**
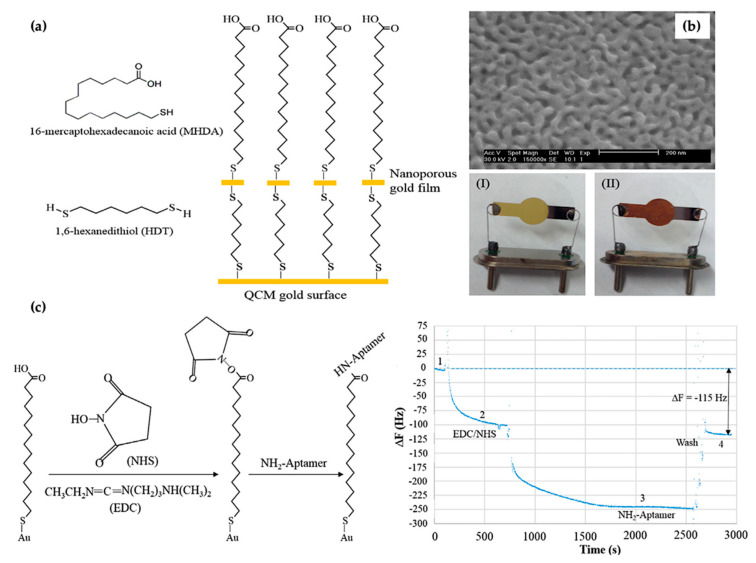
(**a**) Bifunctional dithiol immobilization process onto QCM gold surface; (**b**) SEM image of nanowell-based QCM electrode: (I) the bare QCM electrode and (II) the nanoporous gold film modified QCM electrode; (**c**) design process of NH_2_-aptamer immobilization and QCM aptasensor sensorgram graph: (1) the SAM generated on the sensor in equilibrium with distilled water; (2) NHS/EDC activation; (3) NH_2_-aptamer immobilization; and (4) washing to obtain the baseline. Reprinted with permission from Ref. [115] 2017, Wang.

A label-free QCM aptasensor based on nanowell material for quick and sensitive detection of H5N1 AIV was designed by Wang et al. The design process of the nanowell-based electrode for the nanoporous gold film included immobilization onto the gold electrode surface through a bifunctional dithiol 1,6-hexanedithiol (Figure 5a). For this purpose, a mixture solution was prepared of 1% dithiol 1,6-hexanedithiol (HDT) and 20 mM MHDA (16-mercaptohexadecanoic acid) at a ratio of 1:1. The thickness of the nanofilm was reported as 120 nm. The pore size of the nanoporous film synthesized using a metallic corrosion technique was ~20 nm. After characterization studies, nanoporous film was coated onto a nanowell-based QCM gold chip surface using the SAM technique (Figure 5). Then, the specific aptamer was attached to a QCM aptasensor through covalent bonding. The mechanism of the NH_2_-aptamer immobilization is displayed in Figure 5b. QCM gold electrode characterization was carried out through scanning electron microscopy (SEM). The linear concentration range was obtained from 2^−4^ to 2^4^ hemagglutination units (HAUs)/50 μL. The limit of detection was found to be 2^−4^ HAU/50 μL for AIV H5N1. No signal was observed for non-target AIV subtypes, including H1N1, H2N2, H7N2, and H5N3. The authors reported that further development of this aptasensor could be applied to detect different viruses [115].

Another AIV H5N1 QCM aptasensor platform to enhance the signal produced for detection of the AIV H5N1 was reported by Brockman et al. First, streptavidin was coated onto the QCM electrode’s gold surface after binding biotin-labeled aptamers. Afterwards, QCM aptasensor response was enhanced by adding aptamer-attached magnetic nanoparticles. The magnetic nanoparticles’ amplification of the aptasensor response was effective at low AIV H5N1 concentrations. The LOD value for this aptasensor was calculated as 1 HAU [116].

In another study, a QCM aptasensor for label-free detection of HepBV virus was established by Giamblanco et al. They designed a system for sensing HepBV DNA by immobilizing a thiol-ssDNA aptamer on the surface of the QCM gold electrode. QCM electrode gold surfaces thus functionalized with thiolated ssDNA were characterized using atomic force microscope (AFM) measurement. The QCM aptasensor was able to detect fmol/cm^2^ target HepBV virus with an ssDNA probe without using any amplification steps or labeling method. The authors were able to perform more sensitive determination by controlling the ssDNA density on the electrode surface. They reported that these results facilitated the basic use and portability of the developed POC biosensor device for label-free and quick detection of HepBV [117].

A biotinylated-DNA immobilized QCM aptasensor for detecting hepatitis C virus (HCV) in serum was developed by Skladal et al. The functionalization process of the QCM electrode surface included the immobilization of cysteamine and activation with glutaraldehyde followed by addition of either avidin or streptavidin; 10 MHz AT-cut gold electrode (diameter 5 mm) quartz crystals were used in this work. The authors reported results showing significantly higher immobilization efficiency with avidin as compared to streptavidin. The piezoelectric aptasensor was able to perform real-time monitoring of hybridization in 10 min. The biotinylated-DNA–avidin-immobilized aptasensor was reused 30 times. From the economic point of view, the reusability of QCM aptasensors is quite promising [118].

### 3.2. Bacteria

**Figure 6:**
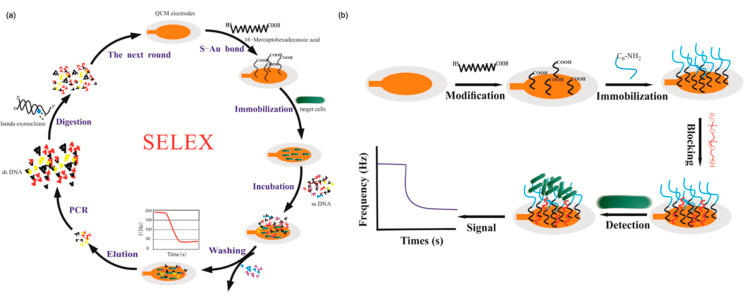
(**a**) SELEX process of DNA aptamers; (**b**) preparation of the QCM aptasensor. Reprinted with permission from Ref. [106] 2017, Wang.

The quick, reliable, accurate, and highly sensitive detection of bacteria is a focus of diverse areas, particularly public health [119]. Therefore, the progress of novel quick, specific, and sensitive biosensors for the determination of pathogens is of remarkable importance [120]. *Salmonella typhimurium* is a pathogen bacteria that causes outbreaks of diseases [121]. *S. typhimurium* infection causes fever, diarrhea, abdominal cramps, and even death. It is important to develop a quick, selective, and sensitive system to detect *S. typhimurium* pathogen bacteria. Wang et al. designed a novel QCM aptasensor for label-free and real-time detection of *S. typhimurium* employing an AT-cut 7.995 MHz quartz crystal gold electrode. The LOD value was calculated as 10^3^ CFU/mL of *S. typhimurium* within one hour [106]. The preparation of the QCM aptasensor process is shown in Figure 6.

*Escherichia coli (E. coli)* O157:H7 infection causes various symptoms including severe abdominal cramps, acute hemorrhagic diarrhea, and hemolytic uremic syndrome. The design of a highly sensitive and specific technique is critically important for controlling outbreaks and disease progression in infected individuals. Yu et al. developed a single-stranded DNA aptamer-attached QCM aptasensor for real-time detection of *E. coli* O157:H7. Whole cells of *E. coli* O157:H7 bacteria were employed using the SELEX process. The detection limit was 1.46 × 10^3^ CFU/mL of *E. coli* O157:H7 within 1 h. The presented results show that the specific ssDNA aptamer chosen by means of whole-bacterium SELEX possesses high affinity [122].

### 3.3. Proteins

**Figure 7:**
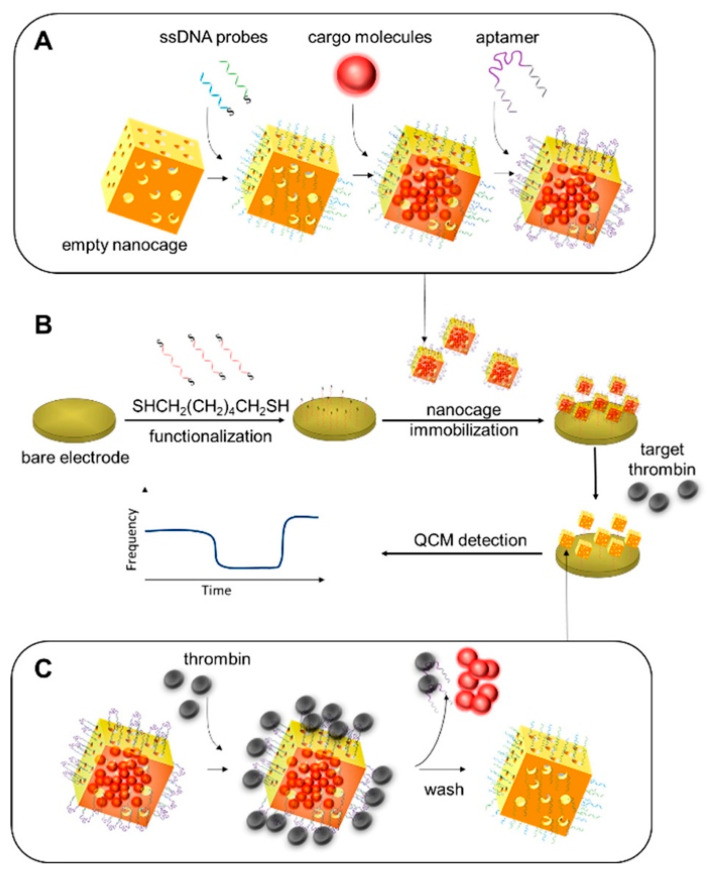
(**A**) Illustration of process of loading cargo molecules into AuNCs; (**B**) preparation of aptasensor for detection of thrombin; and (**C**) thrombin-triggered release of cargo molecules. Reprinted with permission from Ref. [126] Xi.

The detection of proteins can provide valuable information for clinical diagnosis applications [123]. Thrombin is a significant biomarker, and its rapid and selective detection is very important for diagnosis and prevention of related diseases [124,125]. Xi et al. designed a target-triggered delivery of cargo molecules-based QCM aptasensor. Gold nanocages (AuNCs) were characterized using a transmission electron microscope (TEM). Empty nanocages were loaded with ssDNA molecules, capped with specific aptamers, then coated on a QCM chip gold surface for real-time detection of thrombin [126], showing a broad linear concentration dynamic range of 0.0086 nM−86 nM. The LOD values were calculated as 7.7 pM and 1.2 nM in PBS buffer and a human serum sample, respectively. The preparation of this QCM aptasensor design for the detection of thrombin is shown in Figure 7.

Hianik et al. developed a novel QCM aptasensor device for the detection of thrombin. The electrode was covered by DNA aptamers of the electrochemical indicator methylene blue (MB), which was bonded to thrombin They reported that MB can be used to detect thrombin with high sensitivity and selectivity. The lower limit of the detection QCM method was 1 nM [127].

Iijima et al., developed a thrombin-binding DNA-aptamer attached QCM aptasensor. They used DNA-aptamer in their previously developed ~30 nm bio-nanocapsules (ZZ-BNC). ZZ-BNC was modified by replacing the ZZ domain with a DNA-binding single-chain lambda Cro (scCro) domain to expand the versatility of ZZ-BNC. The nanocapsule-coated scCro-BNC-QCM aptasensor chip immobilized with thrombin-binding DNA aptamers showed ~5.5-fold higher thrombin binding capacity and ~6000-fold higher detection sensitivity compared to a QCM aptasensor chip directly coated with DNA aptamers. They reported that the number of bound thrombin molecules per DNA aptamer molecule increased ~7.8-fold with scCro-BNC coating [128].

Deng et al. developed a first-time combined QCM and surface-enhanced Raman spectroscopy (SERS) platform for detection of thrombin. The functionalization process of the QCM electrode included a 1,6-hexanedithiol (HDT) monolayer with gold nanoparticles (20 nm AuNPs) assembled on immobilized HDT with stable Au–S linkage. Then, thiol-modified aptamers were assembled on this electrode surface. AuNPs were used to amplify the frequency signal significantly. The limit of detection for thrombin was 0.1 μM. In the concentration range of 0.1 to 1.0 μM, a good linear correlation was obtained for the determination of thrombin. This presented combination could further develop the progress and application of QCM and SERS in protein analysis with aptamers [129].

Aptasensor platforms for analysis of HIV-1 Tat protein by immobilizing a specific RNA aptamer on a QCM electrode were reported by Tombelli et al. This QCM aptasensor was compared with a surface plasmon resonance (SPR)-based aptasensor. The biotin–avidin linking was immobilized onto the gold surface of the quartz crystal chip. Both devices displayed similar reusability, sensitivity, and specificity. The linear detection range of the QCM was from 0 to 1.25 ppm [130].

Minunni et al. developed a specific RNA aptamer-immobilized QCM aptasensor for the trans-activator of transcription (Tat) protein of HIV-1. In this work, a specific RNA aptamer was utilized as a biological recognition element. The antibody was immobilized on a layer of carboxylated dextran previously deposited on the QCM gold chip surface. The linear range with the antibody was from 0 to 2.5 ppm, and the limit of detection was 0.25 ppm [131].

Yao et al. developed a QCM aptasensor for fast sensing of Immunoglobulin E (IgE) in human serum samples. Aptamers were immobilized non-covalently using a monolayer of avidin on the QCM gold surface. They reported this sensor to be suitable for the detection of IgE within 15 min. It showed a linear detection range between 2.5 μg/L and 200 μg/L in buffer solution and human serum, respectively. This QCM aptasensor was suitably designed for label-free and selective detection of proteins, and represents an innovative device for future proteomics [132].

### 3.4. Cells

**Figure 8:**
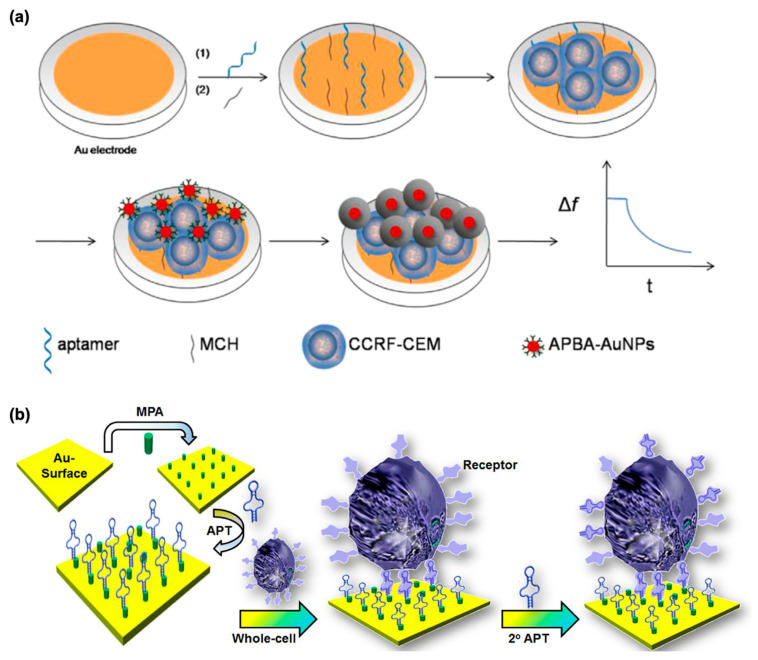
(**a**) Design of QCM aptasensor for detection of leukemia cells, reprinted with permission from Ref. [140] 2014, Shan. (**b**) Capture of HepG2 Cells on aptamer immobilized QCM gold surface electrode, reprinted permission from Ref. [141] 2014, Kashefi-Kheyrabadi.

Recently, investigations of the interaction of whole cells with QCM sensors have been reported [133,134]. Leukemia is one of the most common deadly cancers [135]. It is caused by blood or bone marrow cancer [136]. A sensitive and accurate diagnosis is important for efficient treatment of this disease. The methods employed today for analysis of leukemia cells are polymerase chain reaction [137], flow cytometry [138], and fluorescence measurement [139]. There is a need to produce simple and cost-effective technologies for rapid, label-free, and selective detection of leukemia cells. Shan et al. developed a new method for specific detection of leukemia cells. They first prepared aminophenylboronic acid-modified gold nanoparticles (APBA-AuNPs) which could bind to cell membranes. Then, these APBA-AuNPs were employed for labeling of cells. Signal amplification was achieved via silver enhancement. A good linear relationship was obtained between 2 × 10^3^–1 × 10^5^ cells/mL. The limit of detection was calculated as 1160 cells/mL. This QCM aptasensor offers a quick, rapid, label-free, and cheap technology for sensitive detection of leukemia cells [140]. The QCM detection process is shown in Figure 8a.

In another study, a label-free QCM aptasensor for selective detection of the hepatocellular carcinoma cell line (HepG2) was reported by Kashefi-Kheyrabadi et al. A sandwich architecture was used on the electrode surface. The related HepG2 cells were captured by a TLS11a aptamer covalently attached to a gold surface (Figure 8b). This QCM aptasensor showed a broad linear range between 1 × 10^2^ and 1 × 10^6^ cells/mL, and the limit of detection was 2 cells/mL. The authors reported that this method offers a simple, cheap, and stable technology for sensitive detection of liver cancer as well as other cancel cell [141].

## 4. Conclusions and Future Perspectives

QCMs are highly reliable for sensing the mass of deposited target samples in both liquid and gas matrices. Moreover, they allow real-time monitoring and have relatively low manufacturing and processing costs. These properties make mass-sensitive devices feasible for numerous nanoscale applications such as detection of cells, viruses, antibody interactions, and DNA hybridizations. While QCM biosensors are mostly used owing to their low cost, they are limited to the operating temperature of quartz, ~350 °C, although high-temperature piezoelectric sensors resisting up to 1250 °C have been reported for different applications [68,142]. The limit of detection of a QCM biosensor is lower compared to a surface acoustic wave (SAW). However, QCMs can provide access to physical parameters of samples by measuring the dissipation factor or another equivalent electrical parameter, providing a more detailed analysis of the surface and interactions thereon that is not limited to measurement of the mass per unit area. The viscoelastic and conformational characteristics of a sample are monitored based on the dissipation parameter. The application of the QCM technique in biomedical applications can be very helpful.

Many different biosensors have been developed based on diverse transducers, including optical, electrochemical, and mass-sensitive varieties. Among these techniques, aptamer-based mass-sensitive biosensors have been comprehensively characterized owing to their high sensitivity, high stability, cost-effectiveness, and simplicity of fabrication. It is well known that early determination of diseases and epidemics is essential to ensuring efficient treatment, and aptamers are highly promising biomolecules in this critical area. The selection of aptamers for biorecognition of related viruses, cancer cells, and proteins has already been achieved. Table 1 summarizes different advantages and challenges of the QCM biosensor concept.

When compared to antibodies, aptamers are an especially good match for the recognition of small molecules with high specificity and affinity. Therefore, the improvement of aptamer-based biosensors of macromolecules, even small molecules, could be an efficient way to expand the range of easily measurable analytes. Another potential advantage of aptamer sensors is that they can be stored at variable temperatures and are reusable for certain time periods. Aptamers are thus suitable as miniaturized and portable biosensors that can be kept for extended time periods.

Due to their potential applications, aptamer production has increased significantly over the past few years. Aptamers are convenient for use in biosensors as sensitive and selective recognition elements with a variety of transducer technologies that allow them to be highly sensitive. It is quite beneficial to unite aptamers with QCM as the transducer. Quartz crystal microbalance has emerged as one of the most popular biosensing devices over the past fifteen years. QCM devices are capable of fast, label-free, real-time, and on-site detection of analytes that are of great public health importance, including influenza and hepatitis B virus (HBV), among others, as well as bacteria and proteins. As such, their use in medical diagnostic applications has increased significantly. It is most important that the QCM is evenly distributed over the entire electrode surface when the target analyte is delivered to the recognition surface. The repeatability of measurements made by QCM in practical applications is limited largely by the unevenness of the sensitivity distribution.

In this review, we have examined QCM biosensors and indicated the applicability of establishing aptamer-attached QCM biosensors for quick, high affinity, sensitive, and label-free detection of biological analytes. We have presented and discussed a considerable amount of research on the use of aptamer-based quartz crystal microbalance technology. We have divided these studies into various sections according to analyte and technique, summarizing aptamer-based QCM platform information in Table 1. By developing more aptamers, new aptasensors can be designed which can play a significant role in the development of future diagnostic methods. The design of aptamers with QCM devices has been successful for both quantitative and qualitative medical applications. Aptamers are considered smart receptors for specific binding of target molecules, including viruses, cells, proteins, bacteria, and biomarkers. Several methods have been advanced to transform target molecule−aptamer binding combinations into physically detecTABLE Signals. The progress of QCM biosensing based on aptamers holds great prospects for the development of new medical applications and analytical platforms. Advanced research, particularly that dedicated to the precision, accuracy, and robustness of the reviewed techniques, is needed in this area.

## Figures and Tables

**Table 1:** The advantages and major challenges of QCM biosensors prepared by various techniques.

Method and Materials	Advantages	Challenges	Ref.
**Aptamer**	label-free detection, specific recognition, online, rapid, highly sensitive analysis, simple to functionalize, non-aggregating, very stable in dehydrated form, more resistant to thermal degradation	anchoring to the surface of QCM electrode, low reproducibility, costly	[143,144,145]
**Antibody**	selective affinity to target molecules, sensitive assays, reproducible results,	substantial decrease in bioactivity owing to the denaturation and random orientation, costly production	[146]
**Molecular imprinting polymer (MIP)**	high selectivity to template molecule, long-term storage stability, potential re-usability, cheap	creates wide cavities, template molecule may covalently bound to the polymer, difficult target removal	[147]
**Metal-organic frameworks (MOFs)**	high sensitivity to target, low power consumption, easy modification	Large-scale manufacturing, improved selectivity, enhancing reproducibility, miniaturized manufacturing methods	[148]

## Data Availability

Not applicable.
